# Sickness

**Published:** 1889-09-28

**Authors:** 


### Words of Consolation.

### SICKNESS.

When the Sisters of Bethany sent their appealing message to our Blessed Lord, "Behold, he whom Thou lovest is sick," they expected that Jesus would immediately come and restore their brother to health. It doubtless appeared to them the kindest and most natural course to be taken by One whom we are distinctly told loved Martha, and her sister, and Lazarus. But there was more than one lesson to teach, and more than one learner to be taught. This sickness was to be for the glory of God, that the Son of God might be glorified thereby. Consequently, our Lord abode in the place where He was two days, until He could say to His disciples "Our friend Lazarus is dead, and I am glad for your sakes I was not there, to the intent that ye might believe." This beautiful story, so pathetically told in the eleventh chapter of St. John's Gospel, has been repeated over and over again since then to the present hour. A succession of loving hearts continue to pour forth their agony for the life of some infinitely dear one who is sick unto death, understanding as little as did Martha and Mary of old that Jesus knows far more than they do of their case, and that He is tenderly watching over them from His home in glory. At times the Lord appears to tarry so long that the desponding heart feels there is no comfort left; it must weep at the grave as Mary did. But though He hides His presence from us it is only for a time; He comes the instant we have taken our sharp though salutary lesson to heart. He shows himself brightly when we have learnt to glorify God by patient submission to His holy will. Then, when we lie passive in His hands, realise that all rest out of Him is but weariness, He sheds upon us the peace that passeth all understanding. Thus does He deal with the mourning heart.

And to him who lies helpless week after week, how gently does the Saviour come with healing on His wings, and prepare the soul for renewed life on earth, or for bliss in a glorious eternity. The heart which at first was cold and repining at its lot, melts and glows with love and gratitude for what it can see was mercy in disguise; the impatient spirit has learnt to check its turbulent desires, and own that He doeth all things well: and though life be even then only one long martyrdom of pain, yet none who have ever tasted how gracious the Lord is to those who trust in Him would change this blessed content for all the joys the world can give. The heavier the burden we are bearing, the purer and more perfect is the rest He gives.

The Sisters of Bethany used the strongest term they could think of to induce our Lord to come to their aid; they said, "He whom Thou lovest is sick," and we may use the same words now when asking for help; all the children of God may claim this blessed title; He hath made us His own for ever unless we fall away.

Deep in the heart of pain God's hand hath set
A hidden rest and bliss,
Take as His gift the pain, the gift brings yet
A truer happiness.
God's voice speaks through it all the high behest
That bids His people enter into rest.

